# The Role of Hormonal and Dietary Factors in Pancreatic Carcinogenesis

**DOI:** 10.1155/1993/70454

**Published:** 1993

**Authors:** A. Christopoulou, F. Blanc, H. Joyeux

**Affiliations:** 1 Laboratory of Nutrition and Experimental Oncology University of Montpellier Medical School Montpellier France; 2 Department of Internal Medicine E University of Montpellier Medical School Montpellier France; 3 FACS Cancer Institute Montpellier Cedex 5 34094 France

## Abstract

The pancreatic cancer continues to represent an important problem, as a cancer with extremely poor prognosis.

To date more than 16 chemicals have been shown to induce pancreatic tumors, in animal models. The tumors developed in rats are essentially of acinar type and those in hamsters mostly of ductal type. Many studies proved the direct or indirect role of hormonal and dietary factors in pancreatic cancer. The development of alternative treatments according to biological and biochemical steps of carcinogenesis is available as adjuvant treatment.

We present herein an overview of current experimental and clinical results in order to understand the evolution, histogenesis and biological behaviour of pancreatic cancer.


*HPB Surgery*, 1993, Vol. 6, pp. 141–150
Reprints available directly from the publisher
Photocopying permitted by license only

© 1993 Harwood Academic Publishers GmbH
Printed in the United States of America

### REVIEW ARTICLE

### THE ROLE OF HORMONAL AND DIETARY FACTORS IN PANCREATIC CARCINOGENESIS

A. CHRISTOPOULOU[1,2], F. BLANC[2] and H. JOYEUX[1]

*[(1)]Laboratory of Nutrition and Experimental Oncology, [(2)]Department of Internal Medicine E, University of Montpellier, Medical School Montpellier, France*

*(Received 11 May 1992)*

The pancreatic cancer continues to represent an important problem, as a cancer with extremely poor prognosis.

To date more than 16 chemicals have been shown to induce pancreatic tumors, in animal models. The tumors developed in rats are essentially of acinar type and those in hamsters mostly of ductal type.

Many studies proved the direct or indirect role of hormonal and dietary factors in pancreatic cancer.

The development of alternative treatments according to biological and biochemical steps of carcinogenesis is available as adjuvant treatment.

We present herein an overview of current experimental and clinical results in order to understand the evolution, histogenesis and biological behaviour of pancreatic cancer.

KEY WORDS: Pancreatic carcinogenesis, Experimental models, Hormonal and nutritional factors

### 1. INTRODUCTION

Adenocarcinoma of the pancreas continues to represent an important problem worldwide. It is a disease with an extremely poor prognosis: fewer than 20% of affected patients survive the first year, and only 3% are alive five years after the diagnosis[1].

Despite this grim outcome, considerable progress has been made in our understanding of the pathogenesis of this disease with the lowest five-year survival rate. However, an overview of current experimental and clinical results does offer some promise for the development of more effective drug combinations or combined modality regimens for systemic and local therapy. We present herein the most recent data about the experimental and clinical models of pancreatic carcinogenesis and also some comments about the therapeutic approach to pancreatic cancer.

Address correspondence to: Prof. Henri Joyeux, M.D., FACS, Cancer Institute, 34094 Montpellier Cedex 5, France

Supported by the "French National Committee against Cancer"

### 2. MODELS OF EXPERIMENTAL CARCINOGENESIS IN PANCREATIC CANCER

The malignant tumors of the pancreas in humans are adenocarcinomas from pancreatic ductal cells in 3/4 of the cases. All the other pancreatic cells participate to a more or less important extent in pancreatic carcinogenesis[2,3]. It's well known that the pancreatic tissue contains fewer ductal cells than acinar cells. This means that it is possible that pancreatic cancer is produced by a late cellular transformation of acinar to ductal cells[3]. Different models of pancreatic carcinogenesis were developed *in vivo* and *in vitro*, in order to evaluate the different steps of pancreatic carcinogenesis and the biological processes of the different pancreatic cells during this differentiation[3]. In experimental models under the influence of different carcinogens there is a transformation of acinar cells to ductal cell[3].

In order to understand the evolution, histogenesis and biological behaviour of exocrine pancreatic carcinoma, some reproducible experimental models have been developed in certain rodent species. To date more than 16 chemicals have been shown to induce pancreatic tumors. Although some of these chemicals appear species specific in their effect on the pancreas, others have been shown to be capable of inducing pancreatic tumors in more than one species[4].

Pancreatic tumors can be induced experimentally in animals by lifelong administration of tobacco-specific nitrosamines in drinking water, as well as by parenteral administration of other N-nitroso compounds, such as the N-nitrosobis (2-hydroxypropyl) amine (BPH) and the N-nitroso (2-hydroxypropyl)-2-oxoptopyl amine (HPOP) which are the most potent carcinogens in rat and hamster animal models[1-3]. It has been suggested that these N-nitroso compounds reach the pancreas either through the blood or through refluxed bile that is in contact with the pancreatic duct[1]. These nitrosamines can be administrated by an only intra-venous injection or repeated injections[3].

The delay before the cancer is apparent after the introduction of the carcinogen may vary from one month for the focal histologic lesions, to 4-6 months for lesions > 3mm and finally plus to 1 year for the invasive pancreatic tumor[2].

### *2.1 Carcinogenesis in Rat Models*

The most used compound is the o-diazoacetyl-N serine (azaserine). Injecting as an only dose intraperitoneously (IP) in rats produces DNA alterations during the first hour and these alterations persist for 4 weeks and the DNA repair is completed after 9 weeks. *In vitro* doses of 30 $\mu$g/ml, produce DNA alterations in cellular pancreatic lines[2].

The principal target of azaserine is the acinar cell and the mode of action is the alkylation of DNA. Therefore it is evident that the most frequent histologic pancreatic lesions are nodular acinar cell atypia. These results suggest that the acinar cell, in these models, is the exclusive cell of pancreatic cancer differentiation[2].

The different types of azaserine administration may play an important role in the final result[5,8]. It has been proved that azaserine may play an initiator and/or a promoter role. The incidence of pancreatic carcinomas varying with the total dose[7].

There is a sequence for the genesis of lesions[2]:

— focal acinar cell atypias (diameter $<$ 1mm)
— nodular acinar cell atypias (diametre $>$ 1mm and $<$ 3mm)
— adenomas (diametre $>$ 3mm)
— *in situ* carcinoma
— adenocarcinoma with adipose tissue invasion.

The 7-12-dimethylbenz-(a)anthracene (DMBA) plays a role in the induction of poorly differentiated pancreatic cancer[3]. The 4-hydroxyaminoquinolone-1-oxyde (HAQO) by a single intravenous injection produces pancreatic necrosis in 48 hours with regeneration in 72 hours and we observed most frequently tumors with atypia or adenomas rather than cancers[2].

Nd (N-methyl-N-nitroso-N-carbonyl)-L-ornithine (MNCO) acts directly. It produces nodular acinar cell atypia with pseudoductal structures only occasionally. On the other hand the main disadvantage of this compound is the induction of cancer in other organs[3].

In conclusion pancreatic cancer in the rat animal model is an acinar cell type carcinoma with different zones of cellular atypia[3].

### 2.2 *Carcinogenesis in Hamster Models*

In hamsters, the nitrosamines induce a ductal type pancreatic carcinoma[4]. The most commonly used are POP, its derivative, HPOP, and N-nitroso-2-6-dimethylmorpholine (NOMM) that can methylate pancreatic DNA at the level of guanin and phosphate groups. The first lesions induced are hypertrophy or metaplasia of ductal epithelium. Then papillary structures, similar to those observed in humans appear. MNCO induces focal acinar atypia and further differentiation of the acinar structures to ductal[3].

The cellular origin of the cancers observed in hamsters is probably centro-acinar and ductal with a secondary infiltration of the acini or a differentiation of the acinar cells[9-11]. These cancers do not secrete enzymes and their appearance is similar to human cancers[3].

In conclusion tumors developed in rats are essentially of acinar type and those in hamsters are mostly of ductal type.

### 3. DIRECT OR INDIRECT HORMONAL SIGNALS IN PANCREATIC CARCINOGENESIS

The presence of trypsin, chymotrypsin, or bile-pancreatic juice in the rat intestine suppresses the secretion of exocrine pancreatic enzymes. Conversely, the administration of trypsin inhibitors (TI) or diversion of bile pancreatic juice from the duodenum (pancreaticobiliary diversion) result in an increased secretion of digestive enzymes by the pancreas, an enhanced pancreatic response to stimulation by cholecystokinin (CCK) or caerulein (an amphibian skin polypeptide, structurally and functionally related to CCK[12,13] and pancreatic hypertrophy and hyperplasia[14-16]. The trophic effects on the pancreas obtained by chronic injections of either CCK[17] or synthetic caerulein[13] are generally consistent and appear to be closely associated with increases in DNA, RNA and total protein. This suggests that hormonal stimulation, simultaneously enhances hypertrophy and also mitotic cell division[17,18].

Because the administration of exogenous CCK or caerulein stimulates pancreatic exocrine secretion and also with chronic exposure evokes hypertrophy and hyperplasia of the gland[11,19], it is widely held that the stimulatory actions of trypsin inhibitors or pancreaticobiliary diversion on the pancreas are consistent with negative feed-back regulation of endogenous CCK concentration. This concept, originally proposed by Green *et al.*[20,21] holds that the circulating CCK concentration is inversely related to the concentration or presence of digestive enzymes (most notably trypsin or chymotrypsin) in the lumen of the small intestine. Recent direct experimental evidence for the existence of such an enteral feedback mechanism involved in regulation of pancreatic secretion and growth, has come from experiments employing sensitive and relatively specific bioassay and radioimmunoassay methods and have shown that endogenous circulating CCK levels are elevated in rats after administration of trypsin inhibitors or pancreaticobiliary diversion[22-24].

Feeding rats soy flour, containing active trypsin inhibitors results in enlargement of the pancreas with an increase in synthesis, content and secretion of pancreatic enzymes[25]. Similar responses in the rat pancreas are obtained after repeated injection of CCK[17,26]. It has been suggested that dietary trypsin inhibitor induces the chronic release of massive amounts of CCK from the intestinal wall into the blood stream, and in doing so mediates pancreatic alterations[20].

The effects of dietary soybean trypsin inhibitor (SBTI, Kunitz-type) or repeated injections if 95% pure CCK (CCK-39) on the rat pancreas were investigated in a 10-day experiment. The results indicate that SBTI and CCK-39 mainly exert their effects on the exocrine pancreas in a similar but not identical manner. These findings indicate that the intestinal mucosa contains some other unidentified hormonal factor(s) which exerts a trophic effect on the pancreas and/or potentiates that induced by CCK. It is suggested, therefore, that SBTI is a potent releaser not only of CCK but also of another gastrointestinal factor(s) which is associated with CCK action[27].

This view is supported by Kakade *et al.*[28] who demonstrated that antiproteolytic activity in raw soy flour is only partially responsible for pancreatic hypertrophy in the rat. In fact Solomon *et al.*[13] have already shown that secretin markedly increases the hypertrophic effect of caerulein and modifies the pattern of enzyme changes seen in response to it. The pancreatic hypertrophy and/or hyperplasia, evoked by chronic feeding of rats with camostate (FOY-305, a synthetic guanidino-acid ester, and potent inhibitor of trypsin kallikrein, plasmin, thrombin and phospholipase $A_2$ [29]) or other trypsin inhibitors is believed to be a consequence of elevation of circulating endogenous CCK concentration, resulting from inhibition of trypsin in the duodenum[20-24,30]. Camostate like other trypsin inhibitors, has been shown recently to produce hypertrophy or hyperplasia, or both, when administrated chronically to rats[24,31].

It has been shown recently that the administration of CCK receptor antagonists, proglumide and benzotript fails to fully suppress trypsin inhibitor or pancreaticobiliary diversion-induced pancreatic growth in rats[22,24]. These studies suffer, however, from the fact that both proglumide and benzotript exhibit only weak *in vitro* antagonism or peripheral CCK receptors and also display significant cross-reactivity for gastrin receptors, which limits their usefulness for *in vivo* studies[32]. Furthermore, Yamagushi *et al.*[33] have recently shown that proglumide administration alone results in a slight trophic effect on the rat pancreas *in vivo*. Goke *et al.*[24] have also shown that proglumide failed to fully suppress camostate-induced pancreas growth in rats. These findings suggested that humoral factors in addition to CCK are involved in enteral regulation of the pancreas in camostate-induced pancreas growth[24].

For these reasons the role of CCK in camostate-induced pancreas growth was examined by blocking CCK actions with a specific CCK receptor antagonist L=364,718[34]. This compound, a synthetic benzodiazepine derivative, is a highly selective peripheral CCK receptor antagonist that exhibits an affinity for these receptors that is similar to the affinity exhibited by CCK itself[34]. The observation that treatment with camostate + L-364,718 failed to significantly suppress pancreas weights and DNA and RNA concentrations to level below those of control values does however, suggest the possibility that camostate releases endogenous factors other than CCK that are weakly trophic for the pancreas and that CCK may play a role in the maintenance of pancreatic growth in normal rats[34].

The effect of tetragastrin (structurally similar to CCK) on pancreatic tumors induced by azaserine was investigated in wistar rats and it has been shown that prolonged administration of tetragastrin had little or no influence on the number and size of the carcinogen induced pancreatic lesions, although it caused significantly increased cell proliferation[35]. Bombesin, another pancreaticotrophic peptide, appear to stimulate the growth of preneoplastic cell lesions in the azaserine-rat model[36].

Recent data indicate that sex hormones may play an important role in pancreatic carcinogenesis[37,38]. Effects of sex steroids, on pancreatic carcinogenesis during the early stages, were studied in azaserine treated rats of both sexes. These results showed that estradiol treatment and the drop of testosterone levels caused by castration were highly effective in inhibiting the development and growth of preneoplastic lesions of the pancreas of the rats treated with azaserine. This estradiol effect was dose dependent. Therefore, there is evidence that estrogen may act as an inhibitor and androgen as a promoter in the early stage of pancreatic carcinogenesis in rats[39].

A picture is emerging today of how extracellular signals, including hormones, can act together with one another, and regulate the intracellular events that culminate in cell division. Based on operational criteria mitogenic hormones can be viewed as inducing "competence" or "progression". Both competence and progression factors are necessary for cell division and to be effective *in vitro* must be provided in the correct sequence. If this process is subverted — and clearly there are many stages at which it could — one likely outcome could be uncontrolled cell proliferation. Suspicion that such a failure in hormone circuitry may account, at least in part, for the development of neoplasms has recently been heightened by the observations from many laboratories that tumor cells can secrete and respond to their own "growth hormones" thus releasing their dependence on systemic supply on competence-inducing factors[40].

The most popular view today holds that tumor cell growth is regulated by a combination of endocrinological (systemic), paracrinological (local) and autocrinological (tumor cell derived) hormones.

An imbalance or imbalances in this circuitry, coupled perhaps with the generation of tumor cells that are hyperresponsive to growth promoting agents, are sufficient to support uncontrolled cell proliferation. This means that circulating hormones can be expected to explain only one part of the complex system of carcinogenesis.

### 4. THE ROLE OF DIET IN EXPERIMENTAL PANCREATIC CARCINOGENESIS

Dietary influences on carcinogenesis have been shown in various tissues by both epidemiological and experimental approaches.

Many studies have shown the effects of dietary modification during the post-initiation phase of pancreatic carcinogenesis.

The nutritional factors that could modify pancreatic carcinogenesis are the following:

— The restriction of calories inhibits carcinogenesis[2,5,11].
— Proteins

The rats having a diet rich in proteins (50%) have a lower incidence of cancer. In the contrary diets poor in proteins don't have this effect[5]. In hamsters fed on a low-protein diet, the incidence of pancreatic adenocarcinomas and *in situ* carcinomas was only 13% as compared to 46% in those fed a high protein diet[4].

### *Lipids*

The effect of a diet rich in lipids on pancreatic carcinogenesis is variable[5,42,43]. Dietary unsaturated fat enhances pancreatic carcinogenesis[43]. It has been shown that a diet of 20% unsaturated fat compared to a 20% saturated fat diet or a control diet (5% unsaturated fat) increased the number of acidophilic acinar foci[44].

### *Trypsin Inhibitors*

Research and public attention have been increasingly focussed on the hypothesis that vitamins and micronutrients may decrease the incidence of some cancers of epithelial origin such as adenocarcinomas of the pancreas. A few studies have been reported concerning the possible inhibitory effects of synthetic retinoids on growth of (pre) neoplastic pancreatic lesions induced in rats and hamsters by carcinogens. Depending on both the animal model employed and the sex of the animals, synthetic retinoids have been found to enhance, to inhibit or to have no effect on pancreatic carcinogenesis[45,46]. The conflicting nature of these data may be ascribed to a difference in the types of retinoids used or a difference in the basal diets used, since in most studies impure (natural ingredients) commercial laboratory diets were used, which may vary in the amount of (un) saturated fat they contain.

The effects of vitamins A, C and E on pancreatic carcinogenesis were studied in rats and hamsters maintained on a semipurified diet high in saturated fat (20%) and it is concluded that vitamins A and C have an inhibitory effect on growth of acidophilic foci in rats and incidence of early lesions in hamsters. Vitamin E does not have a potential protective effect on pancreatic carcinogenesis in hamsters[47].

Birt *et al.*[48] found a reduction in pancreatic adenoma incidence by feeding retinoids to female hamsters, whereas in male hamsters an increase in the incidence of adenomas was observed. They found a consistently elevated incidence of pancreatic carcinoma in males in another experiment and concluded that retinoids enhance pancreatic tumor yields in males to a greater extent than in females[49].

Longnecker *et al.*[46,50,51] also demonstrated inhibition of pancreatic carcinogenesis in the azaserine rat model by a number of retinoids and that selenium enhances this inhibition.

A great deal of public and scientific controversy was generated by a report published 10 years ago that coffee consumption could contribute to the development of pancreatic cancer[53]. In a recent comprehensive review, Gordis analyzed 30 epidemiologic studies addressing this relation[53]. He concluded that although certain ecologic and case-control studies suggest a possible increase in risk, only a few of the case-control studies and none of the prospective studies have confirmed a statistically significant association. On the other hand the effect of chronic coffee ingestion on dietary fat promoted pancreatic carcinogenesis was investigated in rats and hamsters, and it is concluded that chronic coffee consumption has an inhibitory effect on dietary fat promoted carcinogenesis by a mechanism which is still unknown[54].

### 5. POSSIBLE THERAPEUTIC APPROACH

The possibility of hormonotherapy was recently proposed for pancreatic cancers on the base of a few clinical studies and experiments[55]. Experimental studies showed an antiproliferative effect of somatostatin and its analogues on the growth of pancreatic cancer. Redding and Schally[56] reported a significant reduction in tumor weight (51%) and volume (67%) in Wistar Lewis rats bearing the acinar pancreatic tumor DNCP-322 after 21-days administration of somatostatin analogue, somatostatin 14 (30 μg, twice daily). In the second experiment, Syrian golden hamsters bearing a ductal adenocarcinoma were treated with Somatostatin 14 (20 μg, twice daily) for 30 days with resultant diminished tumor weight and significantly decreased tumor volume compared with controls.

Upp *et al.*[57] demonstrated inhibition of two human pancreatic cancers (SKI and CAV) maintained as nude mouse xenografts by the administration of Somastotin analogue SMS 201-995 (100 μg/kg, IP, three times daily). SKI is a CCK-receptor-positive tumor whose growth was stimulated by caerulein[58] and CAV is a CCK-receptor-negative tumor whose growth was not stimulated, both were inhibited to a similar degree by SMS 201-995. Tumor growth of SKI and CAV was also inhibited when treatment was delayed 21 days after tumor implantation.

The inhibitory effect of Somatostatin on pancreatic cancer may be due partially to its ability to inhibit the release or action of CCK and secretin. This response could explain the inhibition seen in CAV, which does not possess CCK receptors and is not affected by exogenous caerulein[58].

Other factors (e.g. EGF) may be important in growth of pancreatic cancer. *In vitro* studies demonstrated receptors for Somatostatin and EGF in a cell line from an undifferentiated human pancreatic cancer (MIA PaCa-2)[59]. Epidermal growth factor stimulates *in vitro* growth of this cancer. Somatostatin reverses the stimulatory effect of EGF by activating dephosphorylation of the EGF receptor[60]. Similar results have been found on the pancreatic acinar cell line AR-4-21 of the rat[61]. On the other hand other investigators, have not found receptors for the Somatostatin on human pancreatic adenocarcinomas biopsies[62]. Receptors for sex hormones have been found in pancreatic tumors that justify the role of hormonotherapy using antioestrogenes or LHRH analogues in the treatment of pancreatic cancer[63].

The effects of Cyclosporine (CsA), an immunosuppressive agent, on azaserine-induced pancreatic carcinogenesis in rats, were investigated by Longnecker *et al.*[64] using the short term assays of the quantitation of atypical acinal cell foci. It is concluded that addition of CsA to the diet inhibits the growth of initiated cells to form foci in the pancreas of azaserine-treated rats. This experimental model is useful in analyzing the modifying role of this immunosuppressant in the induction and growth of epithelial cell tumors, such as adenocarcinoma of the pancreas.

The development of alternative treatments according to biological and biochemical steps of carcinogenesis is available as adjuvant treatment in order to prevent the local recurrence or distant metastasis. It may be possible in the future to develop therapeutic strategies for patients with pancreatic cancer that are based upon manipulation of the concentrations or effects of hormones and/or dietary elements in a similar manner to current strategies that are successfully employed in the treatment of patients with breast cancer.

### *References*

1. Warshaw, A. and Del Castillo, C.F. (1992) Pancreatic adenocarcinoma. *N.Engl.J.Med.*, **326** (7), 455–465
2. Vaucher, E. (1990 Effet de deux inhibiteurs trypsiques sur la carcinogenèse du pancréas induite chez le rat. In: *Thèse Faculté de Médecine*, Montpellier, France
3. Moreau, J. (1991) Les Principaux modèles expérimentaux. In: Baumel, H., Huguier, M., *Le cancer du pancréas exocrine*, Springer-Verlag, France
4. Rao, M.S. (1987) Animal models of exocrine pancreatic carcinogenesis. *Cancer Metastasis Rev.*, **6** (4), 665–676
5. Roebuck, B.D., Yager, J.D. and Longnecker, D.S. (1981) Dietary modulation of azaserine induced pancreatic carcinogenesis in the rat. *Cancer Research*, **41**, 888–893
6. Yager, J.D., Roebuck, B.D., Zurlo, J., Longnecker, D.S. Weselcouch, E.O. and Wilpone, S.A. (1981) A single dose protocol for azaserine initiation of pancreatic carcinogenesis in the rat. *Int.J.Cancer*, **28**, 601–606
7. Longnecker, D.S., Roebuck, B.D., Yager, J.D. Lilja, H.S. and Siegmund, B. (1981) Pancreatic carcinoma in azaserine treated rats: induction, classification and dietary modulation of incidence. *Cancer*, **47**, 1562–1572
8. McGuinness, E.E. Morgan, R.G.H., Levison, D.A., Hopwood, D. and Wormsley, K.G. (1981) Interaction of azaserine and raw soya flour on the rat pancreas. *Scand.J.Gastroenterol.*, **16**, 49–50
9. Flaks, B. (1984) Histogenesis of pancreatic carcinogenesis in the hamster: ultrastructural evidence. *Env. Health Perspect.*, **56**, 187–203
10. Scarpelli, D.G., Rao, M.S., Reddy, J.K. (1984) Studies of pancreatic carcinogenesis in different animal models. *Env.Health Perspect.*, **56**, 219–227
11. Longnecker, D.S. (1986) Experimental models of the exocrine pancreatic tumors. In: Go, V.L.W. (ed.) *Biology, pathology and diseases*. Raven Press, New York, pp. 443–458
12. Bonnevie-Nielsen, V. (1981) Effects of caerulein and trypsin inhibitors on the endocrine mouse pancreas. *Acta Endocrinol.*, **96**, 227–234
13. Solomon, T.W., Petersen, H., Elashoff, J. and Grossman, J.M. (1978) Interaction of caerulein and secretin on pancreatic size and composition in rat. *Am.J.Physiol.*, **235** (6), E714–E719
14. Dowling, R.H. Hosomi, M., Stace, H.N. *et al.*, (1985) Hormones and polyamines in intestinal and pancreatic adaptation. *Scand.J.Gastroenterol.*, **20** (suppl 112), 84–95
15. Case, R.M. (1986) Pancreatic growth and adaptation (1986) *Cur.Opinion Gastroenterol.*, **2**, 650–654
16. Jhonson, L.R. (1987) Regulation of gastrointestinal growth. In: Jhonson L.R. (ed.) *Physiology of the gastrointestinal tract*. Raven, New York: pp. 301–303

17. Ihse, I., Arnesjö, B.O. and Lundquist, I. (1976) Effects on exocrine and endocrine rat pancreas of long term administration of CCK-PZ (Cholecystokinin-Pancreozymin) or synthetic octopeptide CCK-PZ. *Scand.J.Gastroenterol.*, **11**, 529–535
18. Mainz, D.L., Black, O. and Webster, P.D. (1973) Hormonal control of pancreatic growth. *J.Clin.Invest.*, **52**, 2300–2304
19. Solomon, T.E. (1981) Regulation of exocrine pancreatic cell proliferation and enzyme synthesis. In: Jhonson, L.R., (ed.) *Physiology of the gastrointestinal tract.* Raven, New York: pp. 873–892
20. Green, G.M. and Lyman, R.L. (1972) Feedback regulation of pancreatic enzyme secretion as a mechanism for trypsin inhibitor-induced hypersecretion in rats. *Proc.Soc.Exp.Biol.Med.*, **140**, 6–12
21. Green, G.M., Olos, B.A., Matthews, G. and Lyman, R.L. (1973) Protein as a regulator of pancreatic enzyme secretion in rats. *Proc.Soc.Exp.Biol.Med.*, **142**, 1162–1167
22. Lee, P.C., Newman, B.M., Praissman, M., Cooney, D.R. and Lebenthal, E. (1986) Cholecystokinin: a factor responsible for the enteral feedback control of pancreatic hypertrophy. *Pancreas*, **1**, 335–340
23. Louie, D.S., May, D., Miller, P. and Onyang, C. (1986) Cholecystokinin mediates feedback regulation of pancreatic enzyme secretion in rats. *Am.J.Physiol.*, **250**, G 252–259
24. Goke, B., Printz, H., Koop, I., *et al.*, (1986) Endogenous CCK release and pancreatic growth after feeding a proteinase inhibitor (camostate). *Pancreas*, **1**, 509–515
25. Dijkhof, J., Poort, S.R., Poort, C. (1977) Effect of feeding soybean flour containing diets on the protein synthesis pattern of the pancreas. *J.Nutr.*, **107**, 1985–1995
26. Johnson, L.R. (1981) Effect of gastrointestinal hormones on pancreatic growth. *Cancer.*, **47**, 1640–1645
27. Temler, R., Dormond, C., Simon, E. and Morel, B. (1984) The effect of feeding soybean thypsin inhibitor and repeated injection of Cholecystokinin on rat pancreas. *J.Nutr.*, **114**(6), 1083–1091
28. Kakade, M.L., Hoffa, D.E. and Liener, I.E. (1973) Contribution of trypsin inhibitors to the deleterious effects of unheated soybeans fed to rats. *J.Nutr.*, **103**, 1772–1778
29. Freise, J., Magerstedt, P. and Schmid, K. (1983) Inhibition of phospholipase $A_2$ by gabexate mesilate, comostate and aprotinin. *Enzyme*, **30**, 209–212
30. Lhoste, E.F., Roebuck, B.D., Longnecker, D.S. (1988) Stimulation of the growth of azaserine-induced nodules in the rat pancreas by dietary camostate (FOY-305). *Carcinogenesis*, **9** (6), 901–906
31. Otsuki, M., Ohki, A., Oxabayashi, Y., Suehiro, I., Baba, S. (1987) Effect of the synthetic protease inhibitor camostate on pancreatic exocrine function in rats. *Pancreas*, **2**, 164–169
32. Loewe, C.J., Grider, J.R., Vlahcevic, Z.R. (1983) Effect of proglumide, a gastrin/CCK receptor antagonist on gastric acid and pancreatic enzyme secretion *in vivo* (abstr.). *Gastroenterology*, **84**, 1232
33. Yamaguchi, T., Tabata, K. and Jhonson, L.R. (1985) Effect of proglumide on pancreatic growth. *Am.J.Physiol.*, **249**, G 294--298
34. Wisner, J., McLaughlin, R., Rich, K., Susumu, D. and Renner, I. (1988) Effects of L-364,718, a new Cholecystokinin receptor antagonist, on camostate-induced growth of the rat pancreas. *Gastroenterology*, **94**, 109–113
35. Tasuta, M., Iishi, H., Baba, M. and Nakaizumi, A. (1990) Effect of tetragastrin on azaserine-induced carcinogenesis in rat pancreas. *Int.J.Cancer*, **46** (3), 489–492
36. Lhoste, E.F., Longnecker, D.S. (1987) Effect of bombesin and caerulein on early stages of carcinogenesis induced by azaserine in the rat pancreas. *Cancer Res.*, **47** (12), 3273–3277
37. Lhoste, E.F., Roebuck, B.D., Brinck-Johnsen, T. and Longnecker, D.S. (1987) Effect of castration and hormone replacement on azaserine-induced pancreatic carcinogenesis in male and female fischer rats. *Carcinogenesis*, **8** (5), 699–703
38. Lhoste, E.F., Roebuck, B.D., Stern, J.E. and Longnecker, D.S. (1987) Effect of orchiectomy and testosterone on the early stages of azaserine-induced pancreatic carcinogenesis in the rat. *Pancreas*, **2** (1), 38–43
39. Sumi, C., Longnecker, D.S., Roebuck, B.D. and Brinck-Johnsen, T. (1989) Inhibitory effects of estrogen and castration on the early stage of pancreatic carcinogenesis in fischer rats treated with azaserine. *Cancer Res.*, **49** (9), 2332–2336
40. Spiliotis, J., Saint-Aubert, B. and Joyeux, H. (1991) Rôle probable des hormones sexuelles et gastrointestinal dans les cancers digestifs. *Bull.Cancer*, **11**, 1007–1011
41. Roebuck, B.D. (1986) Effects of high levels of dietary fats on the growth of azaserine-induced foci in the rat pancreas. *Lipids*, **21** (4), 281–284

42. Appel, M.J., Garderen-Hoetmer, A. and Woutersen, R.A. (1990) Azaserine-induced pancreatic carcinogenesis in rats: promotion by a diet rich in saturated fat and inhibition by a standard laboratory chow. *Cancer Lett.*, **55** (3), 239–248
43. Roebuck, B.D., Kaplita, P.V., Edwards, B.R. and Praissman, M. (1987) Effects of dietary fats and soy bean protein on azaserine-induced pancreatic carcinogenesis and plasma cholecystokinin in the rat. *Cancer Res.*, **47** (5), 1333–1338
44. Roebuck, B.D. (1986) Effects of high levels of dietary fats on the growth of azaserine-induced foci in the rat pancreas. *Lipids*, **21** (4), 281–284
45. Longnecker, D.S., Curphey, T.J., Kuhlmann, E.T., Roebuck, B.D. and Neff, R.K. (1986) Effects of retinoids in N-nitrosobis (2-oxopropyl) amine treated hamsters. *Pancreas*, **1**, 224–231
46. Roebuck, B.D., Baugmgartner, K.J., Thron, C.D. and Longnecker, D.S. (1984) Inhibition by retinoids of the growth of azaserine-induced foci in the rat pancreas. *J.Natl.Cancer Inst.*, **73**, 233–236
47. Woutersen, R. and Van Garderen-Hoetmer, A.M. (1988) Inhibition of dietary fat promoted development of (pre)neoplastis lesions in exocrine pancreas of rat and hamsters by supplemental vitamins A, C and E. *Cancer Lett.*, **41**, 179–189
48. Birt, D.F., Sayed, S., Davies, M.H. and Pour, P. (1991) Sex differences in the effects of retinoids on carcinogenesis by N-nitrosobis (2-oxopropyl) amine in Syrian hamsters. *Cancer Lett.*, **14**, 13–21
49. Birt, D.F., Davies, M.H. and Pour, P. (1983) Lack of inhibition by retinoids of bis (2-oxopropyl) nitrosamine-induced carcinogenesis in Syrian hamsters. *Carcinogenesis*, **4**, 1215–1220
50. Curphey, T., Kuhlmann, E.T., Roebuck, B.D. and Longnecker, D.S. (1988) Inhibition of pancreatic and liver carcinogenesis in rats by retinoid - and - selinium - supplemented diets. *Pancreas*, **3** (1), 36–40
51. Longnecker, D.S., Curphey, T.S., Kuhlmann, E.T. and Roebuck, B.D. (1982) Inhibition of pancreatic carcinogenesis by retinoids in azaserine-treated rats. *Cancer Res.*, **42**, 19–24
52. MacMahon, B., Yen, S., Trichopoulos, D., Warren, K. and Nardi, G. (1981) Coffee and cancer of the pancreas. *N.Engl.J.Med.*, **304**, 630–633
53. Gordis, L. (1990) Consumption of methylxanthine-containing beverages and risk of pancreatic cancer. *Cancer Lett.*, **52**, 1–12
54. Woutersen, R.A., Van Garderen-Hoetmer, A., Bax, J. and Sherer, E. (1989) Modulation of dietary fat-promoted pancreatic carcinogenesis in rats and hamsters by chronic coffee ingestion. *Carcinogenesis*, **10** (2), 311–316
55. Huguier, M., Baumel, H., Houry, S. and Lacaine, F. (1990) Hormonothérapie du cancer du pancréas exocrine. *Gastroentérol.Clin.Biol.*, **14**, 171–174
56. Redding, T.W. and Schally, A.V. (1984) Inhibition of growth of pancreatic carcinomas in animal models by analogs of hyothalamic hormones. *Proc.Natl.Acad.Sci.USA*, **81**, 248–252
57. Upp, J.R., Olson, D., Poston, G.J. *et al.* (1988) Inhibition of growth of two human pancreatic adenocarcinomas *in vivo* by somatostatin analogs SMS 201–995. *Am.J.Surg.*, **155**, 29–35
58. Upp, J.R., Singh, P., Townsend, C.M. and Thompson, J.C. (1987) Predicting response to endocrine therapy in human pancreatic cancer with cholecystokinin receptors. *Gastroenterology*, **92**, 1677 (abst.)
59. Hierowski, M.T., Liebow, C., du Sapin, K. and Schally, A.V. (1985) Stimulation by somatostatin of dephosphorylation of membrane proteins in pancreatic cancer MIA PaCa-2 cell line. *FEBS Lett.*, **179**, 252–256
60. Liebow, C., Hierowski, M. and du Sapin, K. (1986) Hormonal control of pancreatic cancer growth. *Pancreas*, **1**, 44–48
61. Gambillau, C., Tarkiri-Jouti, N., Buscail, L., Viguerie, N., Vidal, C., Vaysse, N. and Susini, C. (1990) Antiproliferative effect of somatostatin: possible involvement of a membrane phosphotyrosine phosphatase. *Digestion*, **46** (suppl), 14
62. Reubi, J.C., Horisberger, V., Essed, C.E., Jeekel, J., Klijn, J.G. and Lamberts, S.W. (1988) Absence of somatostatin receptors in human exocrine pancreatic adenocarcinomas. *Gastroenterology*, **95**, 760–763
63. Lacaine, F., Houry, S. and Huguier, M. (1989) Traitements adjuvants des cancers du pancréas exocrine. *Rev.Prat.*, **39**, 1962–1964
64. Shinozuka, H., Roebuck, B.D. and Longnecker, D.S. (1990) Cyclosporine inhibition of azaserine-induced atypical acinar cell foci in rat pancreas. *Pancreas*, **5** (4), 389–393

*(On invitation from S. Bengmark)*

